# Supplementary material for: Are carbon emissions trading and green financial instruments synergistic? -Comprehensive quantitative research based on content analysis
Source: PLoS One. 2024 Mar 7;19(3):e0298601. doi: 10.1371/journal.pone.0298601 (PMC10919601; doi:10.1371/journal.pone.0298601)
Supplement: S1 Data — (ZIP) [file pone.0298601.s001.zip › Data/地级市Z_F工作报告.zip!/╘╞─╧/╟·╛╕╩╨/└┤╫╘╣1⁄2╓┌║┼í╛┬φ┐╦╩2╛▌═°í┐_2016.docx]

各位代表：

我代表市人民政府，向大会报告工作。请连同《曲靖市国民经济和社会发展第十三个五年规划纲要（草案）》一并审议，并请市政协委员和列席人员提出意见。

　　一、2015年和“十二五”时期经济社会发展回顾

　　2015年，面对国内外经济形势复杂多变、经济下行压力加大等困难和挑战，市人民政府深入学习贯彻习近平总书记系列重要讲话和考察云南重要讲话精神，认真落实党中央、国务院和省委、省政府的决策部署，在市委的坚强领导下，迎难而上、奋力拼搏，统筹抓好稳增长、调结构、强基础、促改革、惠民生等重点工作，实现了经济社会平稳健康发展。市内生产总值同比增长7.4%，规模以上固定资产投资同比增长18.4%，一般公共预算收入同比增长2.1%，社会消费品零售总额同比增长13.1%，城镇和农村常住居民人均可支配收入同比分别增长8.3%、11%，单位生产总值能耗下降3.5%，物价保持基本稳定。

　　主要抓了8个方面的工作：

　　（一）千方百计确保经济止跌回升。强化经济形势研判，出台促进经济平稳健康发展的30条措施，成立稳增长督导组、驻县督查工作组和帮扶组，狠抓稳增长政策落实，逐步扭转了市内生产总值、一般公共预算收入、工业增加值增速下滑的局面。对重点企业实施“一企一策”精准帮扶，兑现失业保险基金稳岗补贴5315万元，降低企业用电、物流成本7.9亿元，减免小微企业税收1.1亿元，促成241户企业获得银行贷款133.2亿元。探索产业链金融封闭运行办法，推动煤焦化行业上下游联动发展。实施“两个10万元”微型企业培育工程，新增小微企业11027户。实施固定资产投资“四集中两严格”制度。争取上级补助资金226.5亿元、专项建设基金 23.2亿元。

　　（二）紧扣市场需求推动产业转型升级。推动煤矿关闭重组，关闭矿井32对，复产复建137对。昆明冶研四氯化硅综合利用等26个项目开工建设，播乐玫瑰精油提取等15个项目加快推进，瑞丰铝业10万吨铝合金棒等23个项目建成投产。完成规模以上工业增加值475.5亿元，同比增长4.5%。新纳规工业企业101户。完成园区基础设施投资47亿元，10个工业园区列入省“10+50”重点支持发展工业园区。建成高标准现代化蓝莓示范和种苗基地2200亩。建成“百千万”畜禽养殖示范场43个，实现畜牧业产值300亿元。新增省级农业龙头企业9个、农民专业合作示范社15个。培育林下经营企业160户、林业生产专业合作社80个。陆良蔬菜冷链物流交易中心等项目建成运营，罗平白龙潭等10个物流园区顺利推进。启动“互联网+农村电子商务”三年行动计划，建成市级电子商务服务中心1个、县级运行服务中心6个、乡镇和村级服务站66个，淘宝“特色中国·曲靖馆”成功上线。引进商业银行3户，2户企业在“新三板”挂牌上市。

　　（三）加大力度补短板强基础。在加快推进综合交通基础设施建设三年攻坚的基础上，启动五大基础设施网络建设五年会战。西石、普宣、昭会、功待高速公路建成通车，宣曲、沾会、江召、东过境高速公路建设进展顺利。沪昆高铁曲靖北站、富源北站建设有序推进。阿岗水库可研报告通过水利部审查，车马碧水库可研报告即将提交水利部审查。新开工骨干水源工程7件、小型病险水库除险加固工程239件，解决了42.4万农村群众饮水安全问题。启动水上飞机机场建设和验证飞行工作。中缅天然气管道昭通支线（曲靖段）一期工程开工建设，建成城市天然气管道36.1公里，新增天然气用户10306户。新增变电容量51万千伏安、输配电线路345.7公里。“宽带中国”专项行动和“智慧曲靖”建设扎实推进。

　　（四）突出四城联创提升城市形象。成功创建国家卫生城市和国家园林城市，被命名为云南省高原体育训练基地。曲靖市城市总体规划进入公示阶段，“多规合一”迈出实质性步伐。启动国家新型城镇化综合试点，推进珠江源大城市十大工程建设，完成6条、启动13条中心城区城市道路综合改造提升和打通断头路工程。新建和改造港湾式公交站台87个，建成城市污水管网138.6公里。开工建设公租房2893套、改造棚户区27244户。撬动住房公积金沉淀资金38亿元，带动商品房销售271万平方米。制定实施《云南省曲靖城市管理条例》配套管理办法，积极推行“网格化”城市管理模式。

　　（五）积极深化改革扩大开放。在县级成立市场监督管理局，实现“一个部门管市场”。建成行政审批网上服务大厅平台和投资项目并联审批平台。市“中介超市”新入驻中介机构24家。在全省率先实现“三证合一”全覆盖。陆良县中型灌区创新建管机制和农业水价综合改革试点得到国务院领导的充分肯定。农村土地承包经营权确权登记颁证试点工作扎实开展，土地承包经营权、林权、活畜抵押贷款成效明显。机关事业单位养老保险制度改革稳步推进。积极参加南博会、滇沪和滇浙经贸交流活动，加强与国内外知名企业的沟通对接，实际到位市外国内资金827.2亿元、利用外资1.4亿美元，同比分别增长68.9%、32.1%。完成外贸进出口总额5.6亿美元，同比增长25.3%。

　　（六）聚焦重点难点抓好民生工程。扎实开展“挂包帮”“转走访”工作，实施整乡整村推进扶贫开发项目107个，推进美丽家园示范点建设226个，完成农村危房改造6.08万户，20万农村贫困人口摆脱贫困。大力推进“8·03”地震会泽灾区恢复重建，民房拆除重建和修缮加固任务基本完成。新建和改扩建标准化公办幼儿园17所、中小学305所，拆除D级校舍42.4万平方米。云南师范大学附属麒麟学校、曲靖一中卓立学校等重点项目扎实推进。教育教学质量稳步提高，高考再创佳绩。市妇幼保健院新院等项目顺利推进，新建和改扩建乡镇卫生院7所、村（社区）卫生服务机构95所。县级公立医院综合改革和县乡医疗服务一体化管理稳步推进。“单独两孩”政策全面落实。被列为第三批国家公共文化服务体系示范区创建城市。发放“贷免扶补”和失业人员小额担保贷款12.5亿元，新增城镇就业3.57万人，城镇登记失业率为3.2%。发放城乡低保、特困人员供养、临时救助和医疗救助资金11.5亿元，新建居家养老服务中心35个、民办养老机构5个，新建和改扩建农村敬老院14所。民族团结示范“十百千万工程”扎实推进。

　　（七）狠抓责任落实加强社会治理。深刻汲取近年煤矿安全生产事故的惨痛教训，落实安全生产五级五覆盖责任体系，深入开展安全生产专项整治和打非治违专项行动，各类事故发生起数、死亡人数、直接经济损失同比分别下降27.2%、9.2%、60.5%。建成省级食品安全示范县2个。加强社会治安综合治理，全面推行“网格化、信息化”服务，群众对社会治安的满意度进一步提升。“六五”普法全面完成。完善社会矛盾排查预警和调处化解综合机制，建立市、县、乡三级视频接访系统，落实领导干部“大接访、大下访”工作制度，群众合理诉求得到有效解决。

　　（八）坚持不懈抓好政府自身建设。“三严三实”和“忠诚干净担当”专题教育扎实开展。建立行政效能明查暗访监督机制，公开曝光135次770人。加强行政监察和审计监督，问责干部297人，行政处分231人，查出和纠正违纪违规资金2.7亿元。制定并公布市级政府部门权力清单和责任清单，实现“一份清单管边界”。预决算信息实现全公开。启动公务用车制度改革。完成公共资源交易1260项、交易总额77.6亿元。自觉接受市人大及其常委会的法律监督、工作监督和市政协的民主监督，认真办理人大代表议案、建议197件和政协提案342件。

　　支持各民主党派、工商联、无党派人士参政议政，支持工会、共青团、妇联等人民团体发挥作用。国防动员、人民防空、优抚安置工作扎实推进，军政军民团结进一步巩固。外事、侨务、统计、广播电视、档案、保密、史志、应急、地震、移民、气象、水文、邮政等工作取得新成绩，妇女、儿童、青少年、慈善、残疾人、红十字等事业取得新进步。

　　各位代表，随着2015年各项工作的扎实推进，“十二五”规划已完成收官。过去的五年，是曲靖经济社会发展很不平凡的五年，是面对各种困难挑战奋力前行的五年，在全市上下的共同努力下，经济社会发展取得了显著成效，为决战决胜全面建成小康社会奠定了坚实的基础。

　　——综合实力显著提升。市内生产总值从“十一五”末的1005.6亿元增加到1630.3亿元，年均增长9.7%。人均生产总值从17000元增加到27044元，年均增长9.1%。一般公共预算收入从72.4亿元增加到118.1亿元，年均增长10.3%。规模以上固定资产投资从701.5亿元增加到1378.8亿元，年均增长20.8%。社会消费品零售总额从232.8亿元增加到502.4亿元，年均增长15.8%。城镇和农村常住居民人均可支配收入分别达27100元、9451元，年均分别增长11.2%、15.8%。

——产业建设扎实推进。三次产业结构比由18.2:52.4:29.4调整为19.5:39.4:41.1。粮食产量稳定在300万吨以上。实施“百千万”畜禽养殖示范场和牛源体系建设，建成标准化规模养殖场2626个。培育农业龙头企业433个、农民专业合作示范社115个，扶持建设精品庄园29个。地方煤矿矿井由466对减少到287对，非煤产业增加值占工业增加值的比重提高10.2个百分点。非公经济增加值占市内生产总值的比重提高6.9个百分点。工业增加值年均增长8.8%。认定国家级高新技术企业59户、省级众创空间3个，新增中国驰名商标6件。第三产业增加值年均增长10.3%。新建、改造网络配送中心50个，被商务部等部委列为全国区域流通节点城市。新增银行业金融机构6家、证券机构4家，金融机构本外币存贷款余额分别达1833亿元、1263亿元，分别是“十一五”末的1.8倍、2倍。旅游、电子商务、养老服务等产业加快发展，被列为全国养老服务业综合改革试点地区。

——城乡基础逐步夯实。完成15个乡镇改设33个街道区划调整工作，实施市级重点小城镇42个，县以上建成区面积达245平方公里，常住人口城镇化率达43.9%，被列为全国新型城镇化综合试点城市。完成交通投资345.9亿元，新增高速公路258公里，新建、改建农村公路1.1万公里，分别是“十一五”的3倍、1.9倍、1.1倍。沪昆铁路六沾二线建成通车，铁路总里程达576公里。宣威支线机场列入国家长江经济带上游机场群规划。完成水利投资184亿元，是“十一五”的3.7倍，新开工建设骨干水源工程36件、小型病险水库除险加固工程586件、“五小水利”工程30万件。改造中低产田地266.9万亩。新增输配电线路1449公里。建成中缅天然气管道曲靖门站，城市天然气管道达246公里。4G网络行政村覆盖率达70%。

——生态环境不断改善。实施重点节能工程100个，淘汰落后产能769.9万吨，单位生产总值能耗累计下降17%。实施“一水两污”项目136个、重金属污染综合治理项目20个，主要污染物排放总量控制在省下达指标内。农业面源污染综合治理扎实推进。深入推进三年城乡绿化攻坚行动，累计造林331.7万亩，森林覆盖率达45%。建成国家级生态乡镇4个、生态村1个，完成40个乡镇的乡村清洁工程。新创建国家4A级景区2个。

——改革开放步伐加快。稳步推进政府职能转变和机构改革，取消和调整行政审批项目129项，保留市级行政审批项目38项、行政许可项目180项。在全省率先建成市级“中介超市”。实现公共资源应进必进、集中交易。国家基本药物制度实现基层公立医疗机构全覆盖，在全省率先实施医疗联合体改革。集体林权制度主体改革全面完成。开放水平不断提高，实际到位市外国内资金和利用外资年均分别增长34.9%、69.3%，外贸进出口总额年均增长20.8%。

——民生保障明显加强。发放“贷免扶补”、小额担保和小企业贷款55.8亿元，扶持自主创业7.9万人，带动就业25.9万人，农村劳动力转移就业和劳务输出62.6万人次。社会保险制度实现全覆盖，在全省率先实施城乡居民大病医疗补充保险。实施市县两级主城区新建中小学、标准化公办幼儿园建设补助政策和市政府优秀骨干教师特殊津贴制度。新建和改扩建标准化公办幼儿园82所、中小学922所。7所中职学校被列为全国改革发展示范学校。新建和改扩建乡镇卫生院65所、村（社区）卫生服务机构570所，新增病床4000余张。兑现新农合大病保险3.1亿元，新农合累计减免83.6亿元。食品药品依法治理能力明显提升。建成市文化体育公园和一批文化惠民工程，成功承办第14届省运会和第10届省残运会。投入各类资金120亿元，实施整乡整村推进扶贫开发项目2366个，扶贫安居工程1.1万户，减少贫困人口65万人。建成城镇保障性住房12.3万套（户），实施农村危房改造和抗震安居工程13.2万户。

　　各位代表！“十二五”全市经济社会发展取得的成绩，是省委、省政府坚强领导的结果，是市委统揽全局、科学决策的结果，是市人大、市政协履职监督、鼎力支持的结果，是全市各级各部门及广大人民群众团结奋进、共同努力的结果。在此，我代表市人民政府，向全市广大干部群众，向人大代表、政协委员、各民主党派、工商联和无党派人士、各人民团体、离退休老干部、社会各界人士，向驻曲部队指战员、武警官兵和中央、省属单位，向所有关心支持曲靖改革发展的同志们，致以崇高的敬意，并表示衷心的感谢！

　　在肯定成绩的同时，我们也清醒地看到，市内生产总值、一般公共预算收入、规模以上固定资产投资、城镇常住居民人均可支配收入4项指标未能完成2015年计划目标，市内生产总值、城镇化率2项指标未能完成“十二五”规划确定的目标。从客观上看，有市场有效需求不足、传统产业产能过剩、新兴产业发展不足等因素影响；从主观上看，我们对经济发展进入新常态的认识不充分，推动稳增长、调结构的措施针对性、有效性不够是主要原因。综合分析，全市经济社会发展仍然面临诸多困难和问题。主要是：经济总量小、发展不充分，作为欠发达地区的状况没有根本改变；对传统产业发展路径依赖性强，现代产业体系构建还需作出艰苦努力；户籍人口城镇化水平不高，基础设施欠账较大；贫困人口多，精准扶贫精准脱贫任务艰巨；就业、教育、医疗等公共服务能力不足；安全生产隐患多、本质安全度低；群众利益诉求日益多元化，全社会法治意识不强，统筹协调各方面利益关系的难度较大；少数政府部门工作人员改革创新精神不够，执行能力不强，“庸政、懒政、怠政”现象不同程度存在。对此，我们要进一步增强责任意识、危机意识、忧患意识，以更大的决心、更实的措施切实加以解决。

　　二、“十三五”时期经济社会发展目标任务

　　“十三五”时期，既是全面建成小康社会的决胜期，也是全面深化改革的攻坚期，更是在跨越式发展路子上走在全省前列的关键期。全市经济社会发展的指导思想是：高举中国特色社会主义伟大旗帜，全面贯彻党的十八大和十八届三中、四中、五中全会精神，以马克思列宁主义、毛泽东思想、邓小平理论、“三个代表”重要思想、科学发展观为指导，深入贯彻习近平总书记系列重要讲话和考察云南重要讲话精神，以及省委九届十二次全会、市委四届七次全会精神，省委、省政府对曲靖“七个走在全省前列”的要求，坚持全面建成小康社会、全面深化改革、全面依法治国、全面从严治党的战略布局，牢固树立创新、协调、绿色、开放、共享发展理念，坚持发展是第一要务，抢抓发展机遇，主动服务和融入国家、省的发展战略，坚持质量效益与规模速度并重、扩增量与调存量并行、创新驱动与发挥特色资源并举，加快发展与安全发展相协调，加快形成引领经济发展新常态的体制机制和发展方式，统筹推进经济建设、政治建设、文化建设、社会建设、生态文明建设和党的建设，坚持不懈抓投资扩内需、抓产业促发展、抓改革添活力、抓开放拓空间、抓扶贫建小康、抓生态优环境、抓安全保民生、抓作风强保障，在闯出跨越式发展路子上走在全省前列。努力把曲靖建成云南经济强市、对内对外开放的重要枢纽城市、特色生态文化旅游城市，确保与全国、全省同步全面建成小康社会，为谱写好中国梦的曲靖篇章奠定更加坚实的基础。

　　“十三五”时期全市经济社会发展的主要目标是：经济保持9%左右的中高速增长，一般公共预算收入年均增长7%，规模以上固定资产投资年均增长20%以上，经济总量和质量效益全面提升。现代产业体系更加完善，新兴产业占市内生产总值的比重提高到30%，产业结构迈向中高端。全社会研发投入占市内生产总值的比重达1.5%，创新驱动能力明显提升。79.91万贫困人口全部脱贫，贫困县全部摘帽，解决区域性整体贫困。发展空间布局得到优化，国家新型城镇化综合试点取得阶段性成果，基础设施五年会战取得全面胜利，常住人口城镇化率达52%。主要污染物排放总量和单位生产总值能耗控制在省下达的指标内，生产生活方式绿色、低碳水平明显提升，森林覆盖率达50%以上。城镇和农村常住居民人均可支配收入年均分别增长10%和11%，就业、教育、医疗、社保等公共服务体系更加健全，公民素质和社会文明程度显著提升。各项改革全面推进，开放型经济加快发展。法治政府基本建成，各方面制度更加完善。

（一）着力构建结构优化、支撑有力的现代产业体系。按照“两型三化”的要求，着力解决产业低端低效的问题，构建可支撑曲靖未来经济社会持续发展的现代产业体系。加快高原特色农业发展。按照龙头企业引领、现代农业基地支撑、三次产业融合、质量品牌保障的要求，打造曲靖现代农业“升级版”。走产、加、销全产业链发展路子，确保粮食稳定增产，加快10大优势特色农产品基地建设，推进肉牛、生猪、水产品精深加工，深度开发小黄姜、银杏、玫瑰、魔芋、核桃下游产品。加快新型工业化发展。改造提升能源、冶金、烟草、化工等传统优势产业，大力发展生物产业、装备制造、新材料、节能环保等新兴产业，努力打造绿色经济示范带和国家重要的能源基地、装备制造业基地。加快现代服务业发展。推动特色生态文化旅游城市和区域流通节点城市建设，推进养老服务业综合改革试点，促进房地产业转型升级，扩大信息、住房、旅游、教育、文化体育、健康养老、家政等领域消费，社会消费品零售总额年均增长10%以上。实施“互联网+”行动计划，加快“智慧曲靖”建设。推动农业生产企业与农产品加工、冷链物流、电子商务等产业组团发展，将曲靖建成全省最大的农产品交易中心和结算中心。促进产业向园区集中集约发展。搭建以企业为主体的市场化融资平台，推动园区基础设施、公共服务和环保设施全覆盖。积极争取国家政策支持，争创国家军民融合创新示范区。推进产城融合发展，打造一批特色鲜明、竞争力强的产业集群。到2020年，工业园区总产值占全市工业总产值的比重达78%以上。提高科技、人才和金融的支撑能力。发挥担保资金和创投引导基金的作用，推进创新链、产业链和资金链“三链融合”，支持核心关键技术创新突破。鼓励众创、众包、众扶、众筹空间发展，加快建成一批国家和省级重点实验室、产业技术创新战略联盟、工程技术研究中心和院士专家工作站，培育一批科技“小巨人”企业。推进金融产权交易平台和农村综合产权交易中心建设，支持曲靖市商业银行在“新三板”上市、曲靖市农村信用联社改制为农村商业银行，鼓励社会资本发起设立民营银行和投资公司，推动融资担保、保险、证券、期货等行业发展壮大，支持企业扩大股权、债券和票据融资规模。完善金融风险防控体系。

（二）着力构建区域协同、城乡协调的空间格局。坚持城市引领、多点发力，优化城乡区域布局，在协调发展中拓展空间，在补齐短板中增强后劲。优化区域发展空间布局。坚持规划的科学性、指导性，强化对城市空间立体性、平面协调性、风貌整体性、文脉延续性的规划和管控，凸显城市特有的地域环境、文化特色、建筑风格等“基因”，彰显城市个性和灵魂。按照全市一张图、一盘棋要求，推进“多规合一”，编制市域国土空间总体规划，落实主体功能区制度。加快珠江源大城市核心区建设，推进麒沾马（经开区）产业集聚区、陆良—师宗—罗平产业集聚区、宣威—富源—会泽产业集聚区建设，加快县域经济特色化发展，构建“一核三片多点”的区域发展新空间。加快新型城镇化发展。围绕建设珠江源山水园林城市目标，按照“北拓、南限、西强、东控”的思路，完成沾益、马龙撤县设区工作，高标准推进海绵城市建设，加大轨道交通、综合管廊等建设力度，完善城市功能，加快麒沾马一体化发展。开展扩权强县和扩权强镇试点，推进罗平、陆良撤县设市工作。加大县城建设力度，建设一批特色小城镇和美丽宜居乡村，推动城乡基础设施共建共享、基本公共服务均衡发展。大力发展城市经济。推进国家新型城镇化综合试点，深化户籍制度改革，建立健全农业转移人口市民化成本分担机制，维护进城落户农民在农村的合法权益，落实市民化待遇，确保农业转移人口留得下、能发展。推动国家文明城市、健康城市、双拥模范城创建工作，提高城市的宜居性、美誉度和影响力。

（三）着力构建互联互通、快捷高效的基础设施网络。围绕经济发展、城镇化推进等需求，强力推动五大基础设施网络建设。建设互联互通的路网。建成宣威至曲靖等10条高速公路，高速公路通车里程达1000公里以上，力争实现绕城高速和环市高速两闭合，县与县之间、国高网与国高网之间互联互通。新建和改建农村公路1.2万公里。沪昆高铁建成通车，积极支持渝昆高铁和南昆铁路复线建设，力争曲靖至弥勒、曲靖至文山等铁路开工建设。建设方便快捷的航空网。积极开展曲靖国际机场建设前期研究和争取工作，建成宣威支线机场和罗平、麒麟、沾益、会泽等水陆飞机起降点，建设滇东通航环线，将曲靖建成全省领先的通用航空基地。建设集约高效的水网。建成阿岗、车马碧水库，启动黑滩河水库建设，完成会泽迤北等8件中型水库、沾益九龙等30件小（一）型水库建设，新增总库容3.5亿立方米、供水能力4亿立方米。实施一批防洪抗旱、节水高效利用工程。建设安全可靠的能源网。全面完成“气化曲靖”三年行动计划，中心城区天然气气化率达70%以上。提高清洁可再生能源比重，构建适应现代产业发展的优质智能型和分布式能源网络。新增变电容量515.3万千伏安，新建、改造输配电线路5129公里，提高供电可靠性。建设高速共享的信息网。推进“三网融合”，构建覆盖城乡、服务便捷、高速畅通、安全可控的新一代互联网基础设施，实现车站、景区、商务楼宇等公共区域免费WIFI全覆盖，力争光纤网络进村入户。

（四）着力构建精准脱贫、持续发展的长效机制。以更加坚决的态度、更加明确的思路、更加精准的举措，坚决打赢脱贫攻坚战。全面落实精准扶贫精准脱贫基本方略。聚焦建档立卡的农村贫困人口，因地制宜、因村施策、因户施法，加快实施“五个一批”工程，确保罗平2016年摘帽、师宗2017年摘帽、富源2018年摘帽、会泽和宣威2019年摘帽，50个贫困乡、475个贫困村全部出列，79.91万贫困人口全部脱贫；到2020年，实现农村贫困人口“两不愁、三保障”，贫困地区农民人均可支配收入增长幅度高于全市平均水平，基本公共服务主要领域指标接近全市平均水平，贫困地区和贫困群众与全市同步全面建成小康社会。着力解决区域性整体贫困。加快推进乌蒙山、滇桂黔石漠化曲靖片区区域发展与脱贫攻坚，实施一批针对性强、示范带动效应好的重大项目和重大工程，解决4个片区县区域性整体贫困。推进民族地区同步发展。坚定不移执行党的民族政策，深入推进民族团结进步示范区建设，发展繁荣民族文化，妥善处理民族宗教关系，实现各民族和睦相处、和衷共济、和谐发展。

（五）着力构建内引外联、充满活力的改革开放新格局。牢固树立曲靖的优势在区位、出路在开放的理念，着力补齐对外交流少、开放程度低、体制机制不活、国内外知名度不高的短板，提升对内对外开放水平。大力发展外向型经济。充分发挥资源、区位优势，积极培育一批具有较强市场竞争力的外向型企业，支持有实力的企业走出去参与国际产能合作，积极承接东部发达地区产业转移，力争建成曲靖综合保税区。外贸进出口总额年均增长15%以上。搭建对外交流合作平台。主动融入国家和云南省区域发展战略，加强与周边地区发展战略的衔接，在融入区域经济发展中谋求更大优势。搭建产能合作平台，以产业发展、市场互补为基础，缔结一批友好城市，探索“共建园区”“飞地经济”。搭建科技、文化、教育交流平台，主动争取国内外知名大学、科研机构等到曲靖办校、设立分支机构，积极组织一批知名国际赛事或巡回赛事，把曲靖建成辐射全国、面向东南亚的优质高原体育训练和竞赛基地。提高招商引资质量。紧扣发展定位，紧盯行业龙头企业，利用行业协会、商会的优势，采取上门招商、以商招商、委托招商等方式，形成招商引资的“磁场效应”。实际到位市外国内资金年均增长12%以上，实际利用外资年均增长10%以上。激发体制机制活力。落实国家和省深化改革的总体部署，坚持问题导向，突出改革重点，加强供给侧结构性改革，深化市场配置要素、行政管理体制、商事制度、投融资体制等领域改革，完善行政审批、市场准入等机制，营造良好的政策环境。

（六）着力构建环境优美、永续发展的生态体系。坚定不移地走绿色低碳循环发展之路，发展绿色经济、建设绿色城镇、倡导绿色生活，争创生态文明排头兵。大力发展循环经济。推进国家循环经济示范市建设，推行企业循环式生产、产业循环式组合、园区循环式改造，建立循环型工业、农业、服务业发展体系，提高资源利用水平，工业固体废物处置利用率达85%以上。倡导绿色消费，推动生活方式和消费模式向简约适度、绿色低碳、文明健康的方式转变。推进“森林曲靖”建设。坚持保护优先、自然恢复为主，实施山水林田库生态保护和修复工程，加大封山育林、人工造林、退耕还林、矿山复绿力度，提升自然生态系统稳定性。新创建一批国家园林县城（城市）、园林城镇。强化生态环境保护。实施珠江源—南盘江生态保护综合利用工程。加快推进生态文明建设“十大工程”，确保主要污染物排放总量持续减少，大气、土壤、水资源保护水平进一步提高。推动县以上城区污水管网、重点集镇和工业园区污水处理设施全覆盖，县以上城区污水处理率达96%以上。加强农村环境综合整治。完善生态文明制度体系，加大环保执法司法联动，严厉打击污染环境、破坏生态等违法犯罪行为。

（七）着力构建制度健全、覆盖城乡的民生保障网。坚守底线、突出重点、注重公平，着力解决人民群众最关心最直接最现实的利益问题，让人民群众共享改革发展成果。努力提高城乡居民收入。坚持创业带动就业，加强就业援助，动态消除城镇“零就业”家庭，城镇登记失业率控制在4.3%以内。推行工学结合、校企合作培养模式，加强职业技能培训。推动居民收入和经济同步增长、劳动报酬和劳动生产率同步提高，逐步缩小收入差距。全力补齐公共服务短板。深化教育综合改革，促进各级各类教育提升水平、协调发展，实现幼儿园行政村全覆盖，基本消除城镇中小学“大班额”现象，职业教育实现集团化发展。实现家庭经济困难学生资助全覆盖。创建国家公共文化服务体系示范区。加强公共卫生服务体系建设，全面推进县级公立医院改革，推广医疗联合体建设模式，实施基层中医药服务能力提升工程。支持社会力量兴办健康服务业。完善基本药物制度，开展按疾病诊断组分类支付方式改革。坚持计划生育基本国策，全面实施一对夫妇可生育两个孩子政策。创建国家食品安全市。推动社会保险由制度全覆盖向人员全覆盖转变，完善职工养老保险个人账户制度，全面实现城乡医保统筹。健全社会保险和社会救助体系，大力发展社会福利事业。加快推进住房保障和供应体系建设，实现城镇常住人口住房保障应保尽保。推动社会治理工作制度化、系统化、常态化，营造安全、稳定、和谐的发展环境。加强社会诚信体系建设。深入开展中国梦和社会主义核心价值观教育。加强未成年人思想道德建设。完善村规民约，弘扬好家风、好家训、乡贤文化，以文明乡风扮靓美丽宜居乡村。大力发展志愿服务事业，推进志愿服务常态化、制度化。实施诚信曲靖建设工程，建立覆盖全社会的信用信息系统。

实现“十三五”跨越式发展是时代赋予我们的重大使命，是全市人民的殷切期盼。我们坚信，通过全市上下的共同努力，一个充满活力、开放包容、宜居宜业、幸福和谐的新曲靖将展现在大家面前！

　　三、2016年工作安排

　　2016年是实施“十三五”规划的开局之年，也是为全面建成小康社会奠定坚实基础的一年。市人民政府要认真贯彻落实党中央、国务院和省委、省政府的决策部署，在市委的坚强领导下，坚决按照“五位一体”总体布局和“四个全面”战略布局，牢固树立并落实创新、协调、绿色、开放、共享的发展理念，努力为“十三五”开好头、起好步。全市经济社会发展的目标建议为：市内生产总值增长8.5%，规模以上固定资产投资增长25%，一般公共预算收入增长6%，社会消费品零售总额增长11%，城镇和农村常住居民人均可支配收入分别增长9%、11%，城镇登记失业率控制在4.3%以内。

　　为实现上述目标，要重点抓好7个方面的工作：

　　（一）保持经济平稳较快增长

　　努力扩大有效供给。围绕去产能、去库存、去杠杆、降成本、补短板，统筹推进供给侧结构性改革。积极稳妥化解过剩产能，主动淘汰落后产能，支持传统产业进行技术改造和设备更新，增加适销对路的产品供给。打通供需通道，采取“以购代建”方式完成公租房建设2248套。实施棚户区改造11600户，确保货币化安置比例达50%以上。推行产业链金融封闭运行办法，鼓励优先使用本地工业产品，下大力气降低企业制度性交易成本、税费负担、社会保险费、财务成本、电力价格和物流成本，帮助企业减轻负担、渡过难关，力争停产减产的规模以上企业减少50%以上。

　　积极增加有效投资。推进省“四个一百”、市“六个一百”重点项目建设。调整优化投资结构，确保产业投资占全社会固定资产投资的32%以上。谋划和筛选一批事关发展全局的重大项目，完善项目储备和滚动实施机制。落实投资项目网上并联审批核准制，加快项目审批进度。大力推广PPP模式，鼓励和引导民间资本通过特许经营、政府购买服务、股权合作等方式参与项目建设。建立专项建设基金3年滚动项目库，积极争取中央专项基金。加快财政资金拨付进度，将有限的资金集中用于重点项目和重点工作，提高财政资金使用绩效。支持有条件的企业上市融资，扩大资本市场直接融资。整合投融资平台，综合利用好政府资源，壮大市级投融资平台实力。

　　加快释放消费新需求。实施“十景百村”旅游工程，重点推动罗平、会泽、沾益等旅游景区提升品质，建成罗平水上飞机起降点，实现麒麟、沾益、会泽水上飞机验证飞行，每个县（市、区）打造2个以上景村融合发展旅游示范点。发展文化体育产业，争取中国之队U19国际男足A级赛等赛事在曲靖举办。编制区域流通节点城市建设规划，加快滇中物流快递园区等10个物流项目建设。扶持电商产业发展壮大，大力培养电商人才，推进营上镇全省乡村新型购物中心建设试点，创建全国电子商务进农村示范县1个，力争实现电子商务成交额40亿元。深入推进国家养老服务业综合改革试点，争取智慧养老项目落地，新建和改扩建农村敬老院16个，发展居家养老服务中心30个以上、民办养老机构10个以上。

　　培育壮大外向型经济。加强经贸交流合作，力争在缔结友好城市方面有大的突破。启动外向型产业基地建设工作，支持企业扩大出口规模，确保外贸进出口总额增长12%以上。完成曲靖综合保税区可研编制等前期工作。精准对接与曲靖产业体系契合度高的国内外知名企业，确保实际到位市外国内资金增长12%以上，项目开工率50%以上，资金到位率20%以上；实际利用外资增长10%以上。

　　（二）推进五大基础设施网络建设

　　加快路网和航空网建设。加快宣曲、江召、东过境、沾会高速公路建设，启动曲陆高速改扩建、兴义至富源、召夸至泸西、威宁至宣威、寻甸至沾益、三宝至长水高速公路建设，做好宣威至富源等高速公路项目前期工作。启动国道326线杉木箐至宣威段改造提升工程。积极支持做好渝昆高铁前期工作，力争完成曲靖至弥勒铁路工可报告，开展曲靖至师宗至文山铁路工可报告编制。推进宣威支线机场前期工作，启动城市轨道交通和城市铁路前期工作，力争尽快开工建设。

　　加快水网建设。确保阿岗水库尽快开工，力争车马碧水库下半年开工，加快黑滩河水库等10件水源工程前期工作，推进31件在建骨干水源工程，新开工沾益九龙水库等6件以上骨干水源工程，确保所有小（二）型病险水库除险加固工程竣工验收。推进曲靖大型灌区续建配套与节水改造工程。

加快能源网建设。建成中缅天然气管道昭通支线（曲靖段）一期工程、陆良支线和83公里城市天然气管道，推进5座天然气加气站建设，启动应急气源储配中心建设，新增居民用户6.6万户。罗平鲁西背靠背直流换流站建成投入使用。新增变电容量56万千伏安、输配电线路405公里。

加快信息网建设和互联网应用。积极落实省“云上云”行动计划，制定“互联网+”行动方案，推动宽带网络普及提速，确保光纤网络、4G移动互联网行政村全覆盖。加快落实与移动、电信、联通、普天、华为等企业的战略合作协议，推进“智慧曲靖”建设。积极推进“两化融合”。整合分散在部门的“孤立数据”，建立互联互通、信息共享的政务服务数据交换平台和无缝对接的电子政务服务系统。

　　（三）提升工业支撑能力

　　实施工业转型升级工程。延伸煤化工产业链，加快煤矿转型升级步伐，力争生产原煤3000万吨；推进煤化与石化耦合项目前期工作，支持煤、焦、化、冶产业上下游联动发展。延伸铝产业链，推进电解铝向铝合金、铝型材、汽车轮毂等下游产业发展。发展壮大装备制造业，启动一汽红塔“三个基地”建设前期工作，推进体育用品生产基地、数控机床等项目建设。大力发展生物产业，加快玫瑰、银杏等深加工项目建设。积极培育新材料产业，推进液态金属研发中心、特种聚酯切片等项目建设。实现工业增加值增长9%。

　　实施工业园区提升工程。完善园区规划，合理布局园区主导产业，严格控制入园标准，提高投资强度，提升园区集聚能力。采取PPP等模式推进园区基础设施建设，完成投资40亿元。加大园区土地收储和开发力度，变“生地招商”为“熟地招商”。加快曲靖经开区产业发展，推进麒麟、沾益、马龙等工业园区空间布局调整和产业转型升级。加快国家军民融合创新示范区前期工作，力争尽快启动建设。

　　实施科技创新和品牌打造工程。制定鼓励科技创新的政策措施，争取曲靖科创企业孵化中心通过国家认定，新认定国家级高新技术企业25户、省级创新型试点企业6户、省级科技型中小企业60户，新创建一批众创空间、科技企业孵化器、院士（专家）工作站。加大“两个10万元”微型企业培育力度，新培育扶持中小微企业4200户、省市级成长型中小企业400户，新纳规工业企业60户以上。推进品牌和标准化发展战略，创建宣威火腿全国知名品牌示范区，力争新增云南名牌3—5个、国家和省级农业标准化示范区4个。

　　（四）扎实推进脱贫攻坚和“三农”工作

　　坚决打好脱贫攻坚战。加快实施“五个一批”工程，全面落实扶贫开发责任、权力、任务、资金“四到县”机制，实施13个省级整乡推进扶贫攻坚项目，完成易地扶贫搬迁26970户，确保罗平县摘帽、10个贫困乡和157个贫困村出列，减少贫困人口17万人。完成“8·03”地震会泽灾区恢复重建年度任务。

　　推动高原特色农业发展。确保粮食播种面积稳定在1000万亩以上。种植蔬菜、水果、花卉、中药材等特色经济作物515万亩。推进蓝莓、猕猴桃、银杏、玫瑰产业4大基地建设，种植蓝莓5000亩，猕猴桃、银杏和玫瑰各1万亩。设立蓝莓产业风险投资基金，加快国家级蓝莓技术研发中心、分选加工、冷链储运、销售中心建设。实施牛源体系工程，推进38个“百千万”畜牧示范场建设。提高农产品精深加工能力，确保“云岭牛”肉牛加工、会泽鲟鱼鱼籽酱等项目竣工投产。控制烟叶总量，提高烟叶质量。引导特色农业拓展生态涵养、观光休闲、文化传承等功能，加快农村一二三产业融合发展。

　　加强农村基础设施建设。改造农村危房6万户以上，打造美丽宜居乡村400个。加强传统村落保护发展工作。实施20个乡镇的乡村清洁工程。完成农田水利基础设施建设20万亩，改造中低产田地48.6万亩。新建、改建农村公路2000公里以上。

深化农村综合改革。推进“三农”金融服务改革创新，创建信用乡镇16个、信用村143个。扎实推进农村土地承包经营权确权登记颁证工作。大力推广陆良水利改革经验，引入社会资本参与水利工程建管运营。全面深化殡葬制度改革。加快培育新型经营主体，新增固定资产投资500万元以上农业龙头企业15个、市级农民专业合作示范社18个、家庭农场200个。

　　（五）加快以人为核心的新型城镇化步伐

　　完善城乡规划体系。认真落实曲靖市城市总体规划，启动麒沾马一体化近期建设、中心城区整体城市设计、综合交通体系等规划编制。开展县城和乡镇规划修编、村镇体系规划工作。完成市规划馆装修布展工作。推进“多规合一”，做好批而未用和闲置土地清理处置工作，继续实施低丘缓坡土地综合利用开发试点，因地制宜推进城镇上山。

　　推进新型城镇化发展。加快推进麒沾马同城化步伐，确保沾益撤县设区，力争马龙撤县设区。推进珠江源大城市十大工程，启动珠江源大道综合管廊、南片区污水处理厂、沾益高铁经济区等项目建设，完成中心城区4条道路改造提升和12条打通断头路工程，建成67.5公里污水管网，确保沪昆高铁曲靖北站、富源北站与正线同步建成。加快20个省级特色小镇、11个市级重点小城镇建设。加快推进国家新型城镇化综合试点，实施城乡统一的户口登记制度，促进农业人口就近就地城镇化，确保常住人口城镇化率提高1.5个百分点。

　　提升城镇管理水平。严格落实《云南省曲靖城市管理条例》及配套实施办法，建设数字化城市管理应用平台，提升精细化、规范化管理水平。推进城市管理体制改革，明确市、县（区）两级职责划分。推动城市共建共管、共治共享，开展噪音、乱停乱放、户外广告等专项整治。

　　（六）大力发展民生事业

　　健全社会保障体系。统筹做好高校毕业生、农村富余劳动力、城乡就业困难群众等重点人群就业工作，扶持1.5万人创业、带动4.5万人就业，农村劳动力转移就业6.5万人，新增城镇就业3.5万人。开展全民参保登记工作。推进城乡医保统筹。落实机关事业单位工作人员养老保险改革政策，健全企业年金办法。完善大病补充医疗保险制度。强化城镇居民医保和新农合基金安全监管。完善以城乡低保为主的社会救助制度，实行低保标准动态调整和社会救助全市统筹机制。加快推进市残疾人康复和职业培训中心项目。

　　办好人民满意教育。大力发展幼儿教育，均衡发展义务教育，推动普通高中多样化发展，促进职业教育产教融合，支持高等教育发展壮大。开展区域校际间校长、教师双向交流试点工作，加快教育信息化建设，扩大优质教育资源覆盖面，确保麒麟、沾益、马龙通过县域义务教育基本均衡国家评估验收。全面改善200所义务教育薄弱学校基本办学条件，确保市县两级主城区11所中小学和47所幼儿园建成投入使用。

　　提升医疗卫生服务水平。深化公立医院改革，加快推进分级诊疗、城乡医疗服务一体化改革，推进县级医院能力提升、乡镇卫生院标准化建设、村级卫生服务全覆盖工程。实施中医药服务能力提升和农村卫生人才素质提升工程。落实一对夫妇可生育两个孩子政策。加快推进市中医院新院和1个县级医院、7个乡镇卫生院、156个村卫生室项目建设，确保市妇幼保健院新院主体工程竣工。

　　加强生态建设和环境保护。推进珠江源—南盘江生态保护综合利用工程，确保珠江源至主城区段开工建设。加快“森林曲靖”建设，完成造林60万亩，森林覆盖率达46%。推进环境监测能力标准化、环境监察执法一体化、环保信息化建设，加强饮用水水源地、万峰湖、北盘江、牛栏江等水资源保护。开展陆良县阎芳河等8条中小河流治理。推进127个“一水两污”和工业园区配套污水处理项目建设，确保市危险废物集中处置中心项目主体工程完工，全面完成主要污染物减排任务。继续推进残膜、化肥、农药、畜禽养殖等污染治理和病死畜禽无害化处理工作。

　　切实做好安全稳定工作。严格落实党委和政府领导责任、部门监管责任、企业主体责任，全面推行安全生产大检查工作机制，建立安全生产巡查制度，突出抓好重点行业领域隐患排查治理，强化打非治违专项行动，坚决杜绝重特大事故，有效控制较大事故，最大限度减少一般事故。加快非煤矿山转型升级工作。完成市级安全生产应急救援基地和安全生产综合调度、煤矿安全监管信息化平台建设。推进基层食品药品安全创建，强化源头治理和全过程监管。继续推进“平安曲靖”建设，完善社会治安防控体系，打好禁毒防艾人民战争，严密防范和依法惩治各种违法犯罪活动。启动“七五”普法工作。完善信访信息平台和网上信访工作制度，扎实推进大交办、大化解、大督查工作，及时就地解决群众合法合理诉求。

　　推进国家公共文化服务体系示范区创建工作。落实党的民族政策，深化民族团结进步示范区创建活动。贯彻党的宗教工作方针，发挥宗教界人士和信教群众在促进经济社会发展中的积极作用。加强国防后备力量建设，做好拥军优属、国防动员、民兵预备役和人民防空工作。统筹做好侨务、地震、档案、保密、地方志、移民开发、社会科学等工作，推进红十字、慈善等事业发展。

（七）努力建设人民满意政府

　　严格依法行政。坚持依法治市、依法行政共同推进，依法设定权力、行使权力、制约权力、监督权力。认真做好行政审批事项接放管工作，全面推行政府权力清单和责任清单制度，实施投资项目“负面清单”管理制度，建设全市统一的投资审批“中介超市”。落实公众参与、专家论证、风险评估、合法性审查、集体讨论决定的重大行政决策法定程序，提高政府规章立法质量，把政府工作全面纳入法治轨道。加强行政执法管理，探索推进综合行政执法，规范行政自由裁量权。推进政务公开常态化，实现决策、执行、结果公开透明，增强政府公信力。配合市人大做好地方立法工作，自觉接受市人大及其常委会的法律监督、工作监督和市政协的民主监督，主动接受人民群众和新闻舆论的广泛监督，提高人大代表议案、建议和政协提案办理质量。

　　坚持务实施政。扎实开展狠抓落实年活动，紧扣责任状、时间表、路线图，强化责任落实和绩效管理，确保确定的目标、制定的政策和作出的承诺落实到位。按照“一个产业、一名领导、一套班子、一抓到底”的模式，建立健全重点产业发展推进机制。办务实的事、开有效地会、发管用的文，完善经济预测预警部门联席会议制度，实行年计划、季分析、月调度工作机制，提高政府抓经济工作的能力和水平。深入推进简政放权、放管结合、优化服务，推进政府职能向创造良好发展环境、提供优质公共服务、维护社会公平正义转变。扎实推进信用体系建设。

　　始终从严治政。坚决贯彻全面从严治党、从严治政各项规定，把党的政治纪律和政治规矩挺在前面，严格落实党风廉政建设责任制，认真履行“一岗双责”，扎实推进政府系统党风廉政建设和反腐败斗争。巩固“三严三实”和“忠诚干净担当”专题教育成果，推进反“四风”常态化和长效化，继续严控“三公”经费，完成公务用车制度改革。完善重点部门和关键岗位廉政风险防控措施，推进公共资金、国有资源资产和领导干部经济责任审计全覆盖，加强公共资源交易管理，把权力关进制度的笼子。加强行政监察，发挥问责倒逼作用，紧盯未落实的事，严查不作为的人，努力营造风清气正、干净干事的良好氛围。

　　各位代表，蓝图已经绘就，行动只争朝夕。让我们在市委的坚强领导下，进一步昂扬斗志、敢于担当，立说立行、真抓实干，不断推动曲靖各项事业向前发展，为全面建成小康社会而努力奋斗！
